# An updated meta-analysis of the asporin gene D-repeat in knee osteoarthritis: effects of gender and ethnicity

**DOI:** 10.1186/s13018-017-0647-3

**Published:** 2017-10-11

**Authors:** Ruoxi Liu, Xueling Yuan, Jing Yu, Qi Quan, Haoye Meng, Cheng Wang, Aiyuan Wang, Quanyi Guo, Jiang Peng, Shibi Lu

**Affiliations:** 1 Institute of Orthopedics, Beijing Key Laboratory of Regenerative Medicine in Orthopedics, Key Laboratory of Musculoskeletal Trauma & War Injuries PLA, Chinese PLA General Hospital, FuXing Road 28th, Beijing, 100853 China; 2 Department of Kampo Medicine, Yokohama University of Pharmacy, 601 Matano-cho, Totsuka-ku, Yokohama-shi, Kanagawa-ken, 245-0066 Japan

**Keywords:** Aspartic acid, Osteoarthritis, Knee, Gender, Ethnicity, Meta-analysis, Polymorphism

## Abstract

**Background:**

Knee osteoarthritis (KOA) is the most prevalent form of knee joint disease and characterized by the progressive degeneration of articular cartilage. Although pathology of KOA remains unknown, genetic factors are considered to be the major cause. Asporin is a group of biologically active components of extracellular matrix (ECM) in articular cartilage, and asporin gene (ASPN) D-repeat polymorphism was reported to be associated with KOA. Thus, our meta-analysis is aimed at investigation of the association between asporin D-repeat polymorphism and susceptibility of KOA.

**Methods:**

We gathered data from MEDLINE, Embase, OVID, and ScienceDirect to search relevant published epidemiological studies through April 2017. Compared with previous studies, our meta-analysis is the first study to investigate the association of ASPN D15, D16, and D17 alleles and KOA susceptibility by ethnic- and sex-stratified subgroup analysis.

**Results:**

We found no significant association between D15 allele and susceptibility to KOA (OR = 1.05, 95% CI 0.95–1.17) in overall population. The same results were observed in the analysis of D16 (OR = 1.01, 95% CI 0.80–1.28) and D17 alleles (OR = 1.28, 95% CI 0.91–1.80). The ethnic- and sex-subgroup analyses did not alter the ORs. However, significant association was detected in the sensitivity analysis of D17 in overall population (OR = 1.05, 95% CI 0.95–1.17) and Asian population (OR = 1.78, 95% CI 1.02–3.11, *P* < 0.05).

**Conclusion:**

Our results indicated that D-repeat polymorphism of ASPN may not play a major role in susceptibility of KOA in ethnic- and sex-specific analysis. Because of the limitations of the present meta-analysis, firm conclusions could not be drawn based on the current evidence, and further studies are required to detect genuine role of ASPN.

## Background

Osteoarthritis (OA), which is characterized by the progressive degeneration of articular cartilage in joints, is one of the most common joint diseases that mainly affects the knees [1, 2]. Joint stiffness and pain appear to be the first symptoms, and joint swelling followed as the result of effusion and synovitis [3]. The knee osteoarthritis (KOA) has been identified of all ages and considered as the most common cause of disability after middle age [4].

However, current therapeutic methods only slow progression of KOA rather than prevent it [5]. The underlying mechanisms of KOA still remain unknown. Epidemiological studies had proved several risk factors associated with KOA, such as age, sex, obesity, kneeling, meniscal injuries, and mechanical forces [4]. Moreover, some previous studies uncovered several genetic linkage and candidate genes correlated with susceptibility to KOA [6, 7]. Taken together, KOA is considered as a polygenic disease controlled by both genetic and environmental factors.

One conventional viewpoint is that KOA is produced by an imbalance between synthesis and degradation of the extracellular matrix (ECM) controlled by chondrocytes [8]. Asporin, which consists of 380 amino acids, belongs to small leucin-rich proteoglycans (SLRPs), a group of biologically active components of ECM in many tissues [9]. Compared with normal cartilage, the expression of asporin is increased in KOA cartilage. It directly binds to transforming growth factor-β (TGF-β) and inhibits tumor necrosis factor (TNF)-β-mediated expression of cartilage genes [10]. The asporin gene (ASPN), which locates in human chromosome 9q22-9q21.3, contains a triplet repeat coding for a polymorphic stretch of aspartic acid residues (D-repeat) in the N-terminal region of the protein [11]. The number of D-repeats varies from 9 (D9) to 20 (D20), and different number of D-repeats may play a different role in KOA onset and development [9, 12]. A positive association between the D14 allele and KOA susceptibility was first reported by Kizawa et al. in a cohort and a case-control study in Japanese population; they also found that D14 allele was upregulated while D13 allele was downregulated in KOA patients [13]. However, relevant meta-analysis yielded inconsistent results. Nakamura et al. reported a positive relation between ASPN D14 allele and KOA susceptibility in Asian population, and results have ethnic differences [14]. But recently, two meta-analyses both demonstrated that ASPN D13 and D14 alleles were not associated with the occurrence of KOA in Asian and European population [15, 16].

There may be two reasons for the abovementioned different results. First, there was a gender difference in the occurrence and pathology of KOA. Women usually have a higher incidence of KOA, and postmenopausal women tend to suffer more severe KOA [17]. Anterior cruciate ligament (ACL) injuries are one of the major causes of KOA in athletes and are more likely to occur in female athletes than in men [18–20]. Besides, it has been reported that females have greater pain intensity, functional limitations, and inflammatory reaction [21, 22]. Second, the incidence and symptoms of KOA have racial differences. Compared with Europeans, the prevalence and severity of KOA are higher in African–Americans [23, 24]. Moreover, Chinese women were reported to suffer more severe radiographic KOA than Caucasian women [25]. In this context, it is reasonable to hypothesize that the association between ASPN and KOA have gender and ethnic differences.

Here, we performed a meta-analysis of recent studies to investigate the association between ASPN D15, D16, and D17 alleles and susceptibility to KOA.

## Methods

### Search strategy and select criteria

We gathered data from MEDLINE, Embase, OVID, and ScienceDirect to search published epidemiological studies through April 2017 that were designed to explore the association between ASPN D-repeat polymorphism and KOA susceptibility. Combinations of keywords used in the search were (“ASPN” or “asporin”), (“polymorphism” or “polymorphisms”), and (“osteoarthritis” or “OA”). No restrictions including languages were imposed on our search.

To be consistent with other previous meta-analysis protocols, we included observational studies that recruited both KOA patients and healthy controls. The diagnostic criteria of KOA should be based on clinical symptoms, radiographic evidence, or joint replacement. Eligible studies should assess the association between ASPN D-repeat polymorphism and KOA susceptibility and had enough genetic frequency to extract. Interim analyses, overlapping study populations, and comparisons of laboratory methods were excluded. Potential studies for the eligibility criteria were reviewed by two independent readers (Liu and Yuan), with a third reviewer (Peng) to settle any discrepancies.

### Date extraction

For all eligible published studies, data were independently extracted from full text with the use of a standard data extraction form by two authors (Liu and Yuan). The standard data extraction form contained information of title, authors, year of publication, study design, sample size, gender, ethnicity, allele count, and allele frequency in KOA patients and healthy controls.

### Statistical analysis

We used the software Review Manager 5.3 (The Nordic Cochrane Center, Copenhagen, Denmark) and STATA 14.0 (Statacorp, College Station, TX, USA) for all the calculations of statistical analysis. The D15, D16, and D17 alleles vs others alleles combined were evaluated respectively because of no specific genotype distribution reported in the included original articles. Thus, we performed meta-analyses of the ASPN D15, D16, and D17 alleles and KOA susceptibility to investigate their association respectively by calculating odds ratios (ORs) and 95% confidence intervals (CIs).

Q-statistic was used to assess between-study heterogeneity, and *P* < 0.1 was considered statistically significant. The recently developed measure *I* ^2^ was also applied to test heterogeneity; values of *I* ^2^ = 25, 50, and 75% were considered low, moderate, and high, respectively [26].

Data are shown as ORs with a 95% CI, and statistical significance was defined as *P* < 0.05 (two-tailed). When heterogeneity was low, the ORs were obtained by fixed effects models [27, 28]. Otherwise, random effects models were used to estimate the ORs [29]. The fixed effects model assumes that genetic factors have similar effects on KOA susceptibility across all included studies and that observed variations among studies result from chance alone [30]. The random effects model assumes that different studies exhibit substantial diversity and assesses both within-study sampling error and between-study variance [29]. A sensitivity analysis was carried out to determine the effect of sample size by omitting one or more studies and assessing the change in the results of the meta-analysis. To test for publication bias, we performed Egger’s linear regression analysis and Begg’s test using the software package STATA 14.0 (Statacorp, College Station, TX, USA) [31].

## Results

### Search results and demographic characteristics

**Fig. 1:**
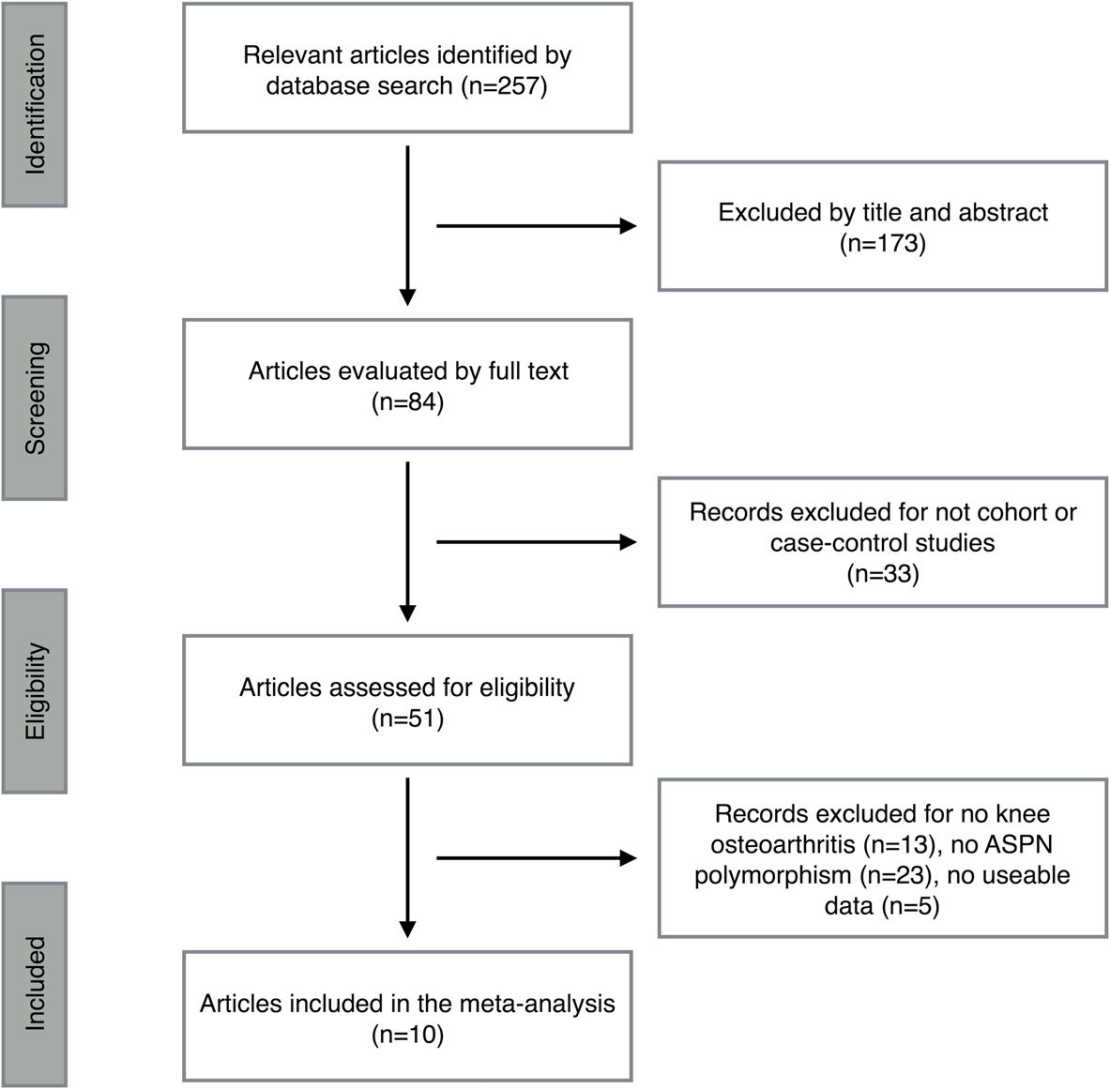
Flow chart of the literature search

The preliminarily literature search yielded 257 articles fulfilling the search criteria of which 51 described case–control studies. Then, we excluded 13 studies for not KOA articles, 23 studies for not ASPN articles, and 5 studies for unusable data. A total of 10 studies fulfilled all inclusion and none of the exclusion criteria (Fig. 1). Noticeably, one of the included articles contained a case–control analysis and a cohort analysis; thus, they were investigated respectively. A total of 11 separate comparisons, with a total of 2745 KOA patients and 3621 controls, which involved 5 Caucasian, 4 Asian, and 2 Latin American populations, were included in this review. Additionally, Of the 11 separate comparisons, 5 sex-stratified comparisons were composed of 1528 females and 1186 males. The recruit criterion of KOA patients was according to symptoms or radiographic evidence in 7 articles and joint replacement in the rest of articles. Characteristics of included articles in the meta-analysis are presented in Table 1.

**Table 1:** Features of the included articles

Study	Ethnicity	Country	Study design	Gender (female/male)		Participants (number)	
				Case	Control	Case	Control
Jiang et al. [37]	Asian	China	Case control	NA	NA	218	454
Kizawa et al. [13]	Asian	Japan	Case control	NA	NA	393	374
Kizawa et al. [13]	Asian	Japan	Cohort	NA	NA	137	234
Song et al. [16]	Asian	Korea	Case control	152/38	154/222	190	376
Atif et al. [36]	Caucasian	USA	Case control	NA	NA	775	511
Jazayeri et al. [35]	Caucasian	Iran	Case control	72/28	72/28	100	100
Kaliakatsos et al. [34]	Caucasian	Greece	Case control	NA	NA	155	190
Mustafa et al. [32]	Caucasian	UK	Case control	158/120	392/356	278	748
Rodriguez-Lopez et al. [33]	Caucasian	Spain	Case control	153/35	115/179	188	294
Arellano et al. [21]	Latin American	Mexico	Case control	130/88	130/92	218	222
González-Huerta et al. [38]	Latin American	Mexico	Case control	NA	NA	93	118
Total				665/309	863/877	2745	3621

*NA* not available

### Results of meta-analysis

**Fig. 2:**
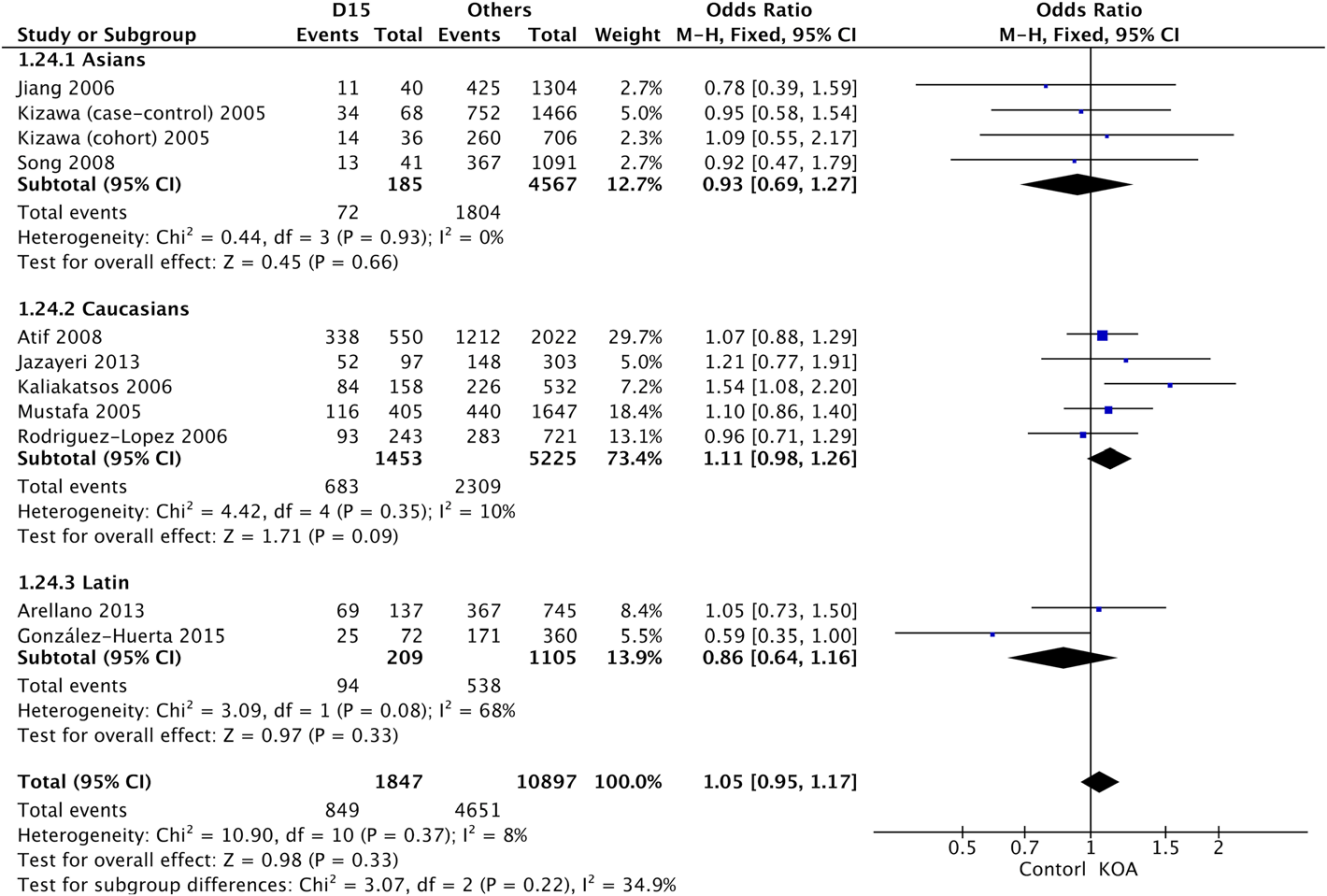
OR and 95% CI for the D15 allele vs. other alleles combined in overall populations and ethnic-specific analysis

**Fig. 3:**
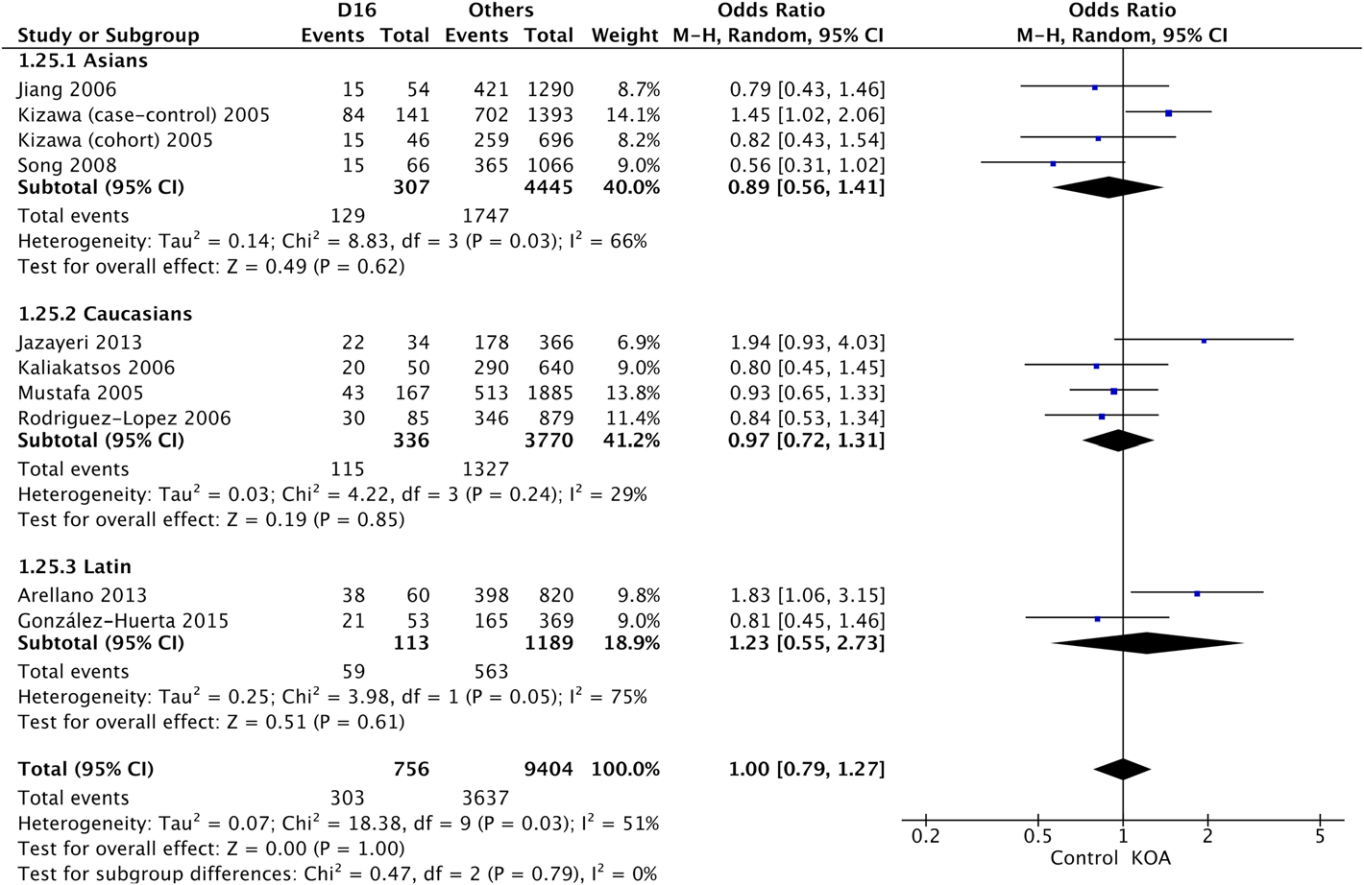
OR and 95% CI for the D16 allele vs. other alleles combined in overall populations and ethnic-specific analysis

**Fig. 4:**
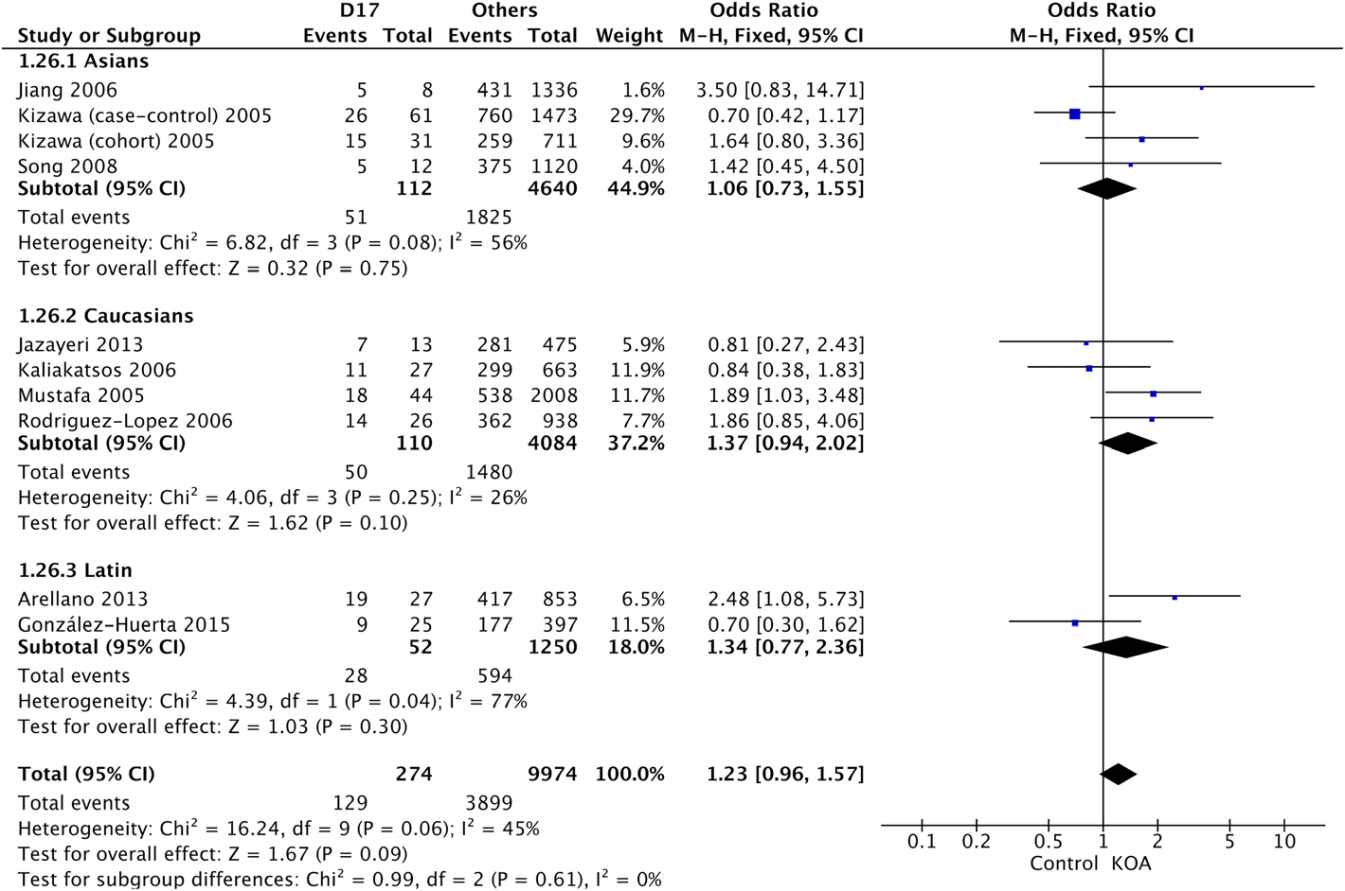
OR and 95% CI for the D17 allele vs. other alleles combined in overall populations and ethnic-specific analysis

Table 2 showed the summary of association between D-repeat polymorphism and susceptibility to KOA. The detailed result is that the pooled OR for the D15 allele vs other alleles combined and its 95% CI included 1 (OR = 1.05, 95% CI 0.95–1.17) (Fig. 2), demonstrating that D15 allele had no significant relationship with susceptibility to KOA in the overall populations included in this review. The results of the combined meta-analysis showed that the D16 allele is not associated with the risk of KOA (OR = 1.01, 95% CI 0.80–1.28) (Fig. 3). The same results were also observed in the analysis of D17 allele (OR = 1.28, 95% CI 0.91–1.80) (Fig. 4), and no significant association was detected between D17 allele and occurrence of KOA.

**Table 2:** Summary ORs, 95% CIs, and heterogeneity of the D-repeat polymorphism and the susceptibility to KOA

Polymorphism	Overall or subgroup (population or gender)	No. of studies	Test of association			Test of heterogeneity		
			OR	95% CI	*P* value	Model	*P* value	*I* ^2^ (%)
D15 versus Others	Overall	11	1.05	0.95–1.17	0.33	Fixed	0.37	8
	Asian	4	0.93	0.69–1.27	0.66	Fixed	0.93	0
	Caucasian	5	1.11	0.98–1.26	0.09	Fixed	0.35	10
	Latin American	2	0.81	0.46–1.42	0.46	Random	0.08	68
	Female	5	1.08	0.87–1.33	0.49	Fixed	0.18	36
	Male	5	0.94	0.71–1.24	0.66	Fixed	0.43	0
D16 versus Others	Overall	10	1.00	0.79–1.27	1.00	Random	0.03	51
	Asian	4	0.89	0.56–1.41	0.62	Random	0.03	66
	Caucasian	4	0.96	0.75–1.22	0.73	Fixed	0.24	29
	Latin American	2	1.23	0.55–2.73	0.85	Random	0.05	75
	Female	5	0.89	0.59–1.34	0.58	Random	0.10	48
	Male	5	0.82	0.56–1.21	0.32	Fixed	0.90	0
D17 versus Others	Overall	10	1.28	0.91–1.80	0.16	Random	0.07	44
	Asian	4	1.30	0.68–2.50	0.43	Random	0.08	56
	Caucasian	4	1.37	0.94–2.02	0.10	Fixed	0.25	26
	Latin American	2	1.32	0.38–4.57	0.66	Random	0.04	77
	Female	5	1.57	0.95–2.59	0.08	Fixed	0.65	0
	Male	5	1.77	0.87–3.60	0.11	Fixed	0.86	0

*NA* not available

**Fig. 5:**
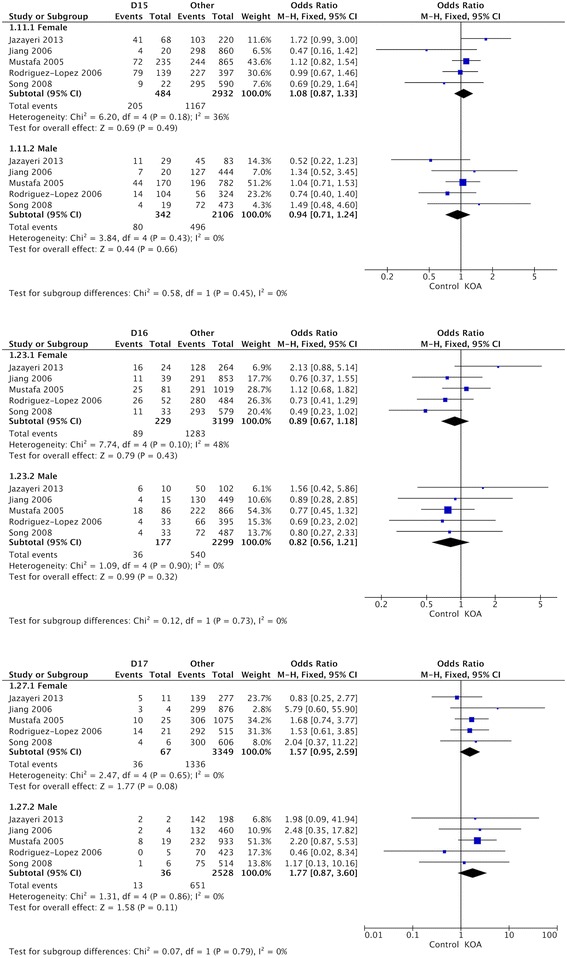
OR and 95% CI for the D15, D16, and D17 alleles vs. other alleles combined in gender-specific analysis

For ethnic-specific analysis, no association was observed between D15 polymorphism and susceptibility to KOA in the Asian (OR = 0.93, 95% CI 0.69–1.27), Caucasian (OR = 1.11, 95% CI 0.98–1.26), or Latin American (OR = 0.81, 95% CI 0.46–1.42) populations (Fig. 5). The same results were also observed in the analysis of D16 and D17 alleles (Fig. 5). Similarly, none of the alleles showed significant sex-specific association with susceptibility to KOA (Fig. 5).

### Sensitivity analysis and publication bias

Because of the high heterogeneity found in D16 and D17 allele analysis for overall and Asian population, we performed the sensitivity analysis to identify the results by removing the case–control study of Kizawa et al. [13] which induced heterogeneity. The results of sensitivity analysis showed the pooled OR of D17 allele vs other alleles combined exceeded 1 (OR = 1.43, 95% CI 1.09–1.89, *P* < 0.05) (Table 3), indicating a significant positive relationship between D17 allele and susceptibility to KOA after sensitivity analysis. The same results were also observed in D17 allele sensitivity analysis for Asian population (OR = 1.78, 95% CI 1.02–3.11, *P* < 0.05) (Table 3). These results differed greatly from previous results without removing the article.

**Table 3:** Sensitivity analysis of the association between D-repeat polymorphism and KOA susceptibility

Polymorphism	Population	Participants(number)		No. of studies	Test of association			Test of heterogeneity		
		Case	Control		OR	95% CI	*P* value	Model	*P* value	*I* ^2^ (%)
D17 versus others	Overall	3237	5398	9	1.43	1.09–1.89	0.01	Fixed	0.26	21
D17 versus others	Asian	1090	2128	3	1.78	1.02–3.11	0.04	Fixed	0.59	0
D16 versus others	Asian	1090	2128	3	0.70	0.50–1.00	0.05	Fixed	0.64	0

Excludes the case–control study of Kizawa et al. [13]

We estimated potential publication bias by Egger’s regression test and Begg’s test. The *P* value for Egger’s and Begg’s test of the asporin D15 allele analysis was 0.354 and 0.213, revealing no proof of publication bias. There was also no significant publication bias in analyses of D16 and D17 alleles (Egger’s and Begg’s test *P* > 0.1).

## Discussion

In the current study, ten published articles (11 comparisons) were included with a total of 2745 KOA and 3621 controls from Caucasian, Asian, and Latin American populations to examine the relationship between ASPN D-repeat polymorphism and KOA susceptibility by ethnic- and sex-specific meta-analysis. We observed that D15, D16, and D17 alleles had no effect on KOA susceptibility with significant heterogeneity in overall population and subgroups mentioned above.

OA is the most common form of joint disorder, leading to physical disability in middle age around the world [4]. The pathogenesis of OA is complex and is still unclear at present, but the effects of genetic polymorphisms on OA susceptibility have attracted increasing attention [6, 7]. Some candidate genes coding for the proteins responsible for the maintenance of articular cartilage have already been reported. Among them, ASPN is an important biologically active component of ECM and a number of evidence demonstrates its role in OA pathogenesis [12]. Patients with OA have an increased expression of ASPN in contrast with healthy controls. One potential mechanism is that ASPN may suppress chondrogenesis by inhibiting TGF-β signaling pathway in the development of OA [10]. The number of D-repeats in N-terminal region of ASPN varies from 9 (D9) to 20(D20) [9]. However, the association between D-repeat polymorphism and KOA susceptibility remains controversial and needs to be further explored.

A positive association (OR = 2.49, 95% CI 1.4–4.4, *P* < 0.01) between the D14 allele and KOA susceptibility was first reported by Kizawa et al. in a cohort sample (394 cases and 374 controls) and a case–control study (137 cases and 234 controls) in Japanese population [13]. However, subsequent studies, which were carried out in different populations worldwide, showed inconsistent results. In UK cases and controls (278 cases and 748 controls), Mustafa et al. reported that ASPN D-repeat polymorphism had little effect on KOA susceptibility (*P* > 0.1) [32]. Another case–control study (188 cases and 294 controls) in Spanish population yielded the same results (*P* > 0.1) [33]. Kaliakatsos et al. indicated that the D15 allele, but not the D14 allele, was found to be associated with increased risk of KOA (OR = 1.54, 95% CI 1.07–2.2, *P* < 0.03) in a Greek case–control study (155 cases and 190 controls) [34]. Similarly, Jazayeri et al. found that D15 allele could be considered as a risk allele only for women in the Iranian population (OR = 1.73, 95% CI 1.01–2.94, *P* < 0.05) [35]. Moreover, in Mexican Mestizo population, D16 allele was observed as a risk factor of KOA in females (OR = 2.226, 95% CI 1.064–3.151, *P* < 0.03), whereas male carriers of D17 allele were more susceptible to KOA (OR = 3.803, 95% CI 1.010–14.317, *P* < 0.05) [21]. There were a number of reasons for these confusing results. Differences in inclusion criteria might be one of them. Some studies recruited KOA patients according to symptoms or radiographic evidence [13, 21, 35–38], whereas the others according to joint replacement [32–34, 39]. Additionally, ethnic and gender differences might be another important reason as well.

Compared with the previous meta-analysis, there are three differences in the present study. First, it is the first study to investigate the association of ASPN D15, D16, and D17 alleles and KOA susceptibility. Second, our report enrolled five sex-stratified studies [32, 33, 35, 37, 39], and it is the first meta-analysis aimed at investigating the association between asporin D-repeat polymorphism and occurrence of KOA in gender-specific approach. Moreover, it included two new references, a new Iranian study [35]and a study in Mexican Mestizo population [38].

In the present meta-analysis, we summarized different studies mentioned above to assess heterogeneity and association between D-repeat polymorphism and KOA susceptibility. The pooling results failed to prove significant associations between the D15, D16, and D17 alleles and KOA occurrence in Caucasians, Asians, and Latin Americans, and the same results were also observed in sex-specific subgroups.

The heterogeneity between separate studies might be caused by lots of complex factors, including age, gender, quality of included studies, diagnostic criteria, inclusion criteria, racial differences, and environmental factors. In our present study, ethnic- and sex-specific subgroup analyses were performed to reduce heterogeneity. In addition, a sensitivity analysis was performed to evaluate the stability of the association between the D16 and D17 alleles and KOA susceptibility in overall and subgroups. After omitting the case–control study of Kizawa et al. [13], a positive association was observed between the D17 allele frequency and increased susceptibility to KOA with low heterogeneity. And similar result was observed in the ethnic-stratified subgroup that D17 allele was a risk factor of KOA in Asians. These opposite results indicated that the pooling results may include a type II error, or false negative, and were lacking enough stability to come to the firm conclusion on association between the D17 allele and KOA susceptibility in overall and Asians group. However, due to the small sample size of D17 allele, this result should be considered with caution.

Several previous studies had demonstrated the gender differences for patients in allele frequencies [34–36, 39]. Although more sex-stratified studies were required because original data of each research cannot be acquired, our study was the first meta-analysis stratified by both gender and ethnicity. The present meta-analysis of the asporin gene D-repeat may not describe a significant difference in sex-specific subgroups, but they reduced the heterogeneity to a certain extent. Nevertheless, the heterogeneity of our study could not be appropriately solved, and some underlying reasons might account for the current predicament.

For instance, different inclusion criteria could be an essential factor for heterogeneity. The included studies were four studies recruiting patients undergone total knee arthroplasty (TKA) [32–34, 39], and six studies enrolled participants by assessing clinical and radiologic evaluation [13, 21, 35–38]. Consequently, the current meta-analysis may include patients with various pathological and radiographic grades. Moreover, the existence of heterogeneity might also be explained by genotype–environment interaction that reflecting different genes respond to environmental variations in different ways.

The limitations of the present meta-analysis were briefly discussed in the following section. First, despite the subgroup analyses performed by ethnicity and gender, the existence of heterogeneity could not be totally resolved. We were failed to apply more types of subgroup analysis stratified by personal conditions or clinical variables because lack of enough original data from included studies. Therefore, the result of our meta-analysis should be interpreted with caution. Second, the environmental and genetic factors remained unclear in the present study. Both of them were responsible for the heterogeneity as well. Third, insufficient including studies with enough raw data, particularly in ethnic- and gender-stratified analyses, influenced the statistical efficacy and caused the high heterogeneity. It is also the reason why meta-regression analysis cannot be performed and was failed to detect the cause of heterogeneity. To settle the problems mentioned above, further studies with more different ethnic populations, unified inclusion criteria, functional research of ASPN D-repeat, sufficient raw data, and environmental and genetic interaction are clearly needed.

## Conclusion

In conclusion, we carried out a meta-analysis of the association between D-repeat polymorphism of ASPN and susceptibility to KOA in Asian, Caucasian, and Latin American populations. Overall, we found no significant relationship between KOA susceptibility and the D15, D16, or D17 alleles. However, results with significant heterogeneity lacked sufficient stability to provide an accurate conclusion on this study. Further studies are required to investigate the role of ASPN; these findings may help to elucidate the pathogenesis of OA and may inform the development of novel therapeutic strategies for OA.

## Abbreviations

ACL: Anterior cruciate ligament
ASPN: Asporin gene
CI: Confidence interval
ECM: Extracellular matrix
KOA: Knee osteoarthritis
OA: Osteoarthritis
OR: Odds ratio
SLRPs: Small leucin-rich proteoglycans
TGF: Transforming growth factor
TKA: Total knee arthroplasty
